# Genetic influences on central and peripheral nervous system activity during fear conditioning

**DOI:** 10.1038/s41398-022-01861-w

**Published:** 2022-03-08

**Authors:** G. Kastrati, J. Rosén, M. Fredrikson, X. Chen, R. Kuja-Halkola, H. Larsson, K. B. Jensen, F. Åhs

**Affiliations:** 1 Department of Clinical Neuroscience, Karolinska Institutet, SE-171 77 Stockholm, Sweden; 2 Department of Psychology and Social Work, Mid Sweden University, SE-831 25 Östersund, Sweden; 3 Department of Biomedical Data Sciences, Leiden University Medical Center, Leiden, the Netherlands; 4 Department of Medical Epidemiology and Biostatistics, Karolinska Institutet, SE-171 77 Stockholm, Sweden; 5 Department of Medical Sciences, Örebro University, SE-701 82 Örebro, Sweden

**Keywords:** Human behaviour, Learning and memory

## Abstract

Fear conditioning is an evolutionarily conserved type of learning serving as a model for the acquisition of situationally induced anxiety. Brain function supporting fear conditioning may be genetically influenced, which in part could explain genetic susceptibility for anxiety following stress exposure. Using a classical twin design and functional magnetic resonance imaging, we evaluated genetic influences (*h*^*2*^) on brain activity and standard autonomic measures during fear conditioning. We found an additive genetic influence on mean brain activation (*h*^*2*^ = 0.34) and autonomic responses (*h*^*2*^ = 0.24) during fear learning. The experiment also allowed estimation of the genetic influence on brain activation during safety learning (*h*^*2*^ = 0.55). The mean safety, but not fear, related brain activation was genetically correlated with autonomic responses. We conclude that fear and safety learning processes, both involved in anxiety development, are moderately genetically influenced as expressed both in the brain and the body.

## Introduction

Threat responses can be elicited by environmental cues through Pavlovian fear conditioning, whereby a neutral stimulus predicts the occurrence of an aversive event [1]. This may be an etiological mechanism for the acquisition of post traumatic disorder [2] and situationally elicited anxiety disorders [3, 4]. Fear conditioning can be studied experimentally in the laboratory by comparing autonomic responses to a fear cue paired with an aversive event with responses to a safety stimulus, that is never coupled with an aversive event [5]. Expression of conditioned fear can mimic memory encoding in anxiety and post-traumatic stress disorders. Thus, using strict experimental control, fear conditioning can elucidate genetic influences on the neural signature of acquisition processes relevant for PTSD and anxiety [6].

The genetic contribution to neural functions supporting fear conditioning in humans is a growing area of research. Several studies have investigated genetic associations with autonomic and brain responses during fear conditioning or extinction of conditioned fear using a candidate gene approach. Single nucleotide polymorphisms related to genes encoding receptors and enzymes expressed in brain have generally been examined [7–10]. Although these studies may hold promise in revealing genetic pathways involved in conditioned fear, the sample sizes have so far been limited and results have been mixed. A recent example is fear conditioning studies on variants of the single-nucleotide polymorphism in the fatty acid amino hydrolase (FAAH) gene (rs324420), where one variant encodes an FAAH enzyme with reduced catabolic efficacy. This single-nucleotide polymorphism has been found to predict extinction in some [11] but not all studies [12], or populations within studies [13]. Hence, although human candidate gene studies provide some evidence for a genetic influence on fear conditioning, results are still not conclusive. In other species, several lines of evidence underpin a genetic contribution to fear conditioning. First, selective breeding studies indicate that rodents can be bred for increased excitability to conditioned threat [14]. Second, silencing or deletion of specific genes can also modulate fear conditioning by inducing changes in the neural circuitry supporting fear conditioning [14]. Third, fear conditioning is an evolutionary conserved type of learning, because it can be observed across a large variety of species, ranging from nematodes [15, 16] and mice [17] to humans [6]. Moreover, there is correspondence across species in terms of neural circuitry supporting fear conditioning [18–20]. This could indicate that findings of genetic influences on brain function in animal models could transfer to humans. Indeed, genetic influences on autonomic conditioned responses have been reported in humans [21], although the contribution of genetic factors to brain functions supporting fear conditioning in our species remains to be established.

To estimate genetic effects on brain function, we adopted a twin design with monozygotic (MZ, *n* = 56 pairs) and dizygotic (DZ, *n* = 67 pairs) twin pairs. Functional magnetic resonance imaging (fMRI) was used to determine blood-oxygen-level-dependent (BOLD) responses during fear conditioning, where one virtual character presented on a computer screen was paired with an electric shock (fear cue, CS+), while another was not associated with shock (safety cue, CS−). Skin conductance responses (SCRs) were measured to index the autonomic component of the conditioned response. The additive genetic influence on brain activity was estimated at every voxel (11) in areas previously shown to be consistently activated by fear conditioning in a comprehensive meta-analysis [6]. The additive genetic influences on mean fear and safety-related activations within the previously defined areas were also evaluated. Finally, the genetic correlation between mean brain activity and SCRs was determined.

## Materials and methods

### Participants

Participants were recruited through the Swedish Twin Registry [22]. There are 87,000 twin pairs in the registry. A total of 3021 were invited to participate by mail, out of which 646 signed up for participation. Individuals were excluded if they were unable to undergo magnetic resonance imaging, had ongoing substance abuse, or were currently enrolled in psychological or pharmacological treatment for psychiatric disorders. Only same sex twin pairs were included and after initial screening, 305 participants underwent fMRI. After data collection, participants were further excluded from the analysis based on excessive head motion if 50% of brain volumes exceeded .5 mm framewise displacement (*n* = 16); or missing data/incomplete twin pair (*n* = 25). The final sample included 62 MZ pairs (35 female, 27 male) and 70 DZ pairs (40 female, 30 male) with a mean age of 33.45 years (SD = 10.22 years, range 20–58 years). All participants provided written informed consent in accordance with the Uppsala Ethical Review Board Guidelines. Participants received reimbursement of SEK 1000 (roughly equal to 100 USD) for their participation.

### Experimental Design

Participants performed three tasks in the MR-scanner, one of which was the fear conditioning paradigm used here. They also performed an interpersonal space task and the Hariri face-matching task. Results from these tasks will be presented elsewhere. For the fear conditioning paradigm, two virtual characters served as CSs and were presented on a screen in the MR scanner at a distance of 2.7 m (Supplementary Fig. S1). The experimental design is similar to our previous reports [23, 24]. One of the characters served as fear cue (CS+) and predicted the unconditioned stimulus (US) whereas the other virtual character served as safety cue (CS−). We refer to fear learning as increased responses to the CS+ compared to the CS−. Safety learning was defined as increased responses to the CS− relative to the CS+. It could however be noted that safety learning also can be assessed by using other types of experimental paradigms, as for example in conditioned inhibition experiments [25]. Fear and safety cues were counterbalanced. Each of the cues appeared for 6 s. An inter-stimulus interval followed each cue for 8–12 s. Prior to the experiment, participants were told they could learn to predict the US yet not told which character who served as the fear cue. Prior to the conditioning phase, each cue was presented four times without reinforcement during a habituation phase. During this phase, no learning had yet occurred. During fear conditioning, the fear and safety cues were displayed 16 times respectively. Eight of the fear cue presentations co-terminated with presentation of the US (50% reinforcement schedule). Four stimulus presentation orders were used to counterbalance cues across subjects. The total time for the conditioning task was 9 min and 47 s.

The shock US was delivered to the subjects’ wrist via radio-translucent disposable dry electrodes (EL509, BIOPAC Systems, Goleta, CA), calibrated before running the experimental task using an ascending staircase procedure so that the shocks were rated as ‘aversive’ [24]. US duration was 16 ms. Shock delivery was controlled using the STM100C module connected to the STM200 constant voltage stimulator (BIOPAC Systems, Goleta, CA).

### Stimuli and Contexts

Two male 3D virtual humanoid characters were created in Unity (version 5.2.3, Unity Technologies, San Francisco, CA) (Supplementary Fig. S1). Contexts and stimuli were presented on a flat screen in the MR scanner with the help of projector (Epson EX5260). The computer running the stimulus presentation used a custom version of Unity (version 5.2.3, Unity Technologies, San Francisco, CA) and communicated with BIOPAC (BIOPAC Systems, Goleta, CA) through a parallel port interface. The software for the parallel port interface was custom made and used standard.NET serial communication libraries by Microsoft (Microsoft Corporation, Albuquerque, New Mexico).

### Brain imaging

Brain imaging data were acquired using a 3.0 T scanner (Discovery MR750, GE Healthcare) and an 8-channel head-coil. T1-weighted structural images were acquired with TR = 2.400 ms, TE = 28 ms, flip angle 11^o^. Functional images were acquired using gradient echo-planar-imaging (EPI) with an interleaved ascending order with TR = 2400 ms, TE = 28 ms, flip angle = 80^o^, slice thickness 3.0 mm^3^ with no spacing, axial orientation, and frequency direction (R/L).

### Skin conductance responses

Skin conductance recording was controlled with the MP-150 BIOPAC system (BIOPAC Systems, Goleta, CA). Radio-translucent disposable dry electrodes (EL509, BIOPAC Systems, Goleta, CA) were coated with isotonic gel (GEL101, BIOPAC Systems, Goleta, CA) and placed on the palmar surface of the left hand. The signal was high-pass filtered at 0.05 Hz and SCRs were scored using Ledalab software package [26] implemented in Matlab 2018 (Mathworks, Inc., Natick, MA). SCRs were analyzed using the maximum phasic driver amplitude 1–4 s after CS onset for each participant. SCRs were range-corrected by dividing all SCRs for each participant with each participant’s average SCR [27].

### Statistical analysis

#### Preprocessing and statistical analysis of fMRI Data

Analyses of fMRI-data were performed using SPM12 (Wellcome Department of Cognitive Neurology, University College, London). Preprocessing of images was done using interleaved slice time correction, realignment, co-registration to acquired T1 image, spatial normalization to Montreal Neurological Institute (MNI) space, and spatial smoothing with an 8 mm Gaussian kernel. First-level analysis used event-related modeling of CS+ and CS− trials as well as US. Stimuli were modeled with duration 6 s with a column vector in the design matrix for each stimulus type. Regressors were convolved with the hemodynamic response function. Anatomic labeling was performed using the SPM Anatomy toolbox v.2.2c [28]. The experimental contrasts were modeled as fear cue greater than safety cue, denoted as fear learning, and safety cue greater than fear cue, denoted as safety learning. The level of significance was set to *P* < 0.05 family-wise error corrected (FWE). Outliers were identified with using the median absolute deviation (MAD) method [29] (see Supplementary Materials).

#### Statistical analysis of SCR

We analyzed SCR data using t-test in JASP [30]. Heritability estimates of autonomic conditioning were calculated using the mets package [31, 32] implemented in *R* [33]. Prior to the genetic modeling, we identified outliers in our data sample using MAD [29] (see Supplementary Materials).

#### Estimation of genetic influences on brain function

Phenotypic variance can be decomposed into additive genetics (A), common or shared environment (C) and unique environment, or error (E) [34]. The A, C, and E-factors are estimated by contrasting MZ-twin pair correlations with DZ-twin pair correlations. The A-factor may be identified as MZ-twins are genetically identical while DZ-twins share 50% of their co-segregating alleles on average. Additionally, we assume that a shared environmental contribution (C) is equally shared within pairs regardless of whether they are MZ- or DZ-twins. Finally, any variance not attributable to factors shared between twins (A and C), i.e., that make twins in pairs dissimilar, are estimated as an E-factor. The genetic influence, *h*^*2*^, can be interpreted as the proportion of a phenotypic variance explained by additive genetics. In the present study, we computed heritability using the APACE (Accelerated Permutation Inference for the ACE model) software [35]. APACE uses a non-iterative linear regression-based method based on squared twin-pair differences, with permutation-based multiple testing correction to control the FWER. APACE takes as input a mask within which it computes heritability based on first-level contrast images. Here, the first-level contrast images represent brain activation for the fear cue greater than the safety cue (fear learning) and the safety cue greater than the fear cue (safety learning). To constrain our analysis to a priori determined brain networks, heritability estimates of fear and safety learning were computed within the two brain masks related to fear and safety learning described in a previous meta-analysis of neuroimaging studies of fear conditioning [6]. Because the common environment often is negligible in twin studies, we compared the goodness of fit of the ACE model to an AE model, excluding the C component, using the likelihood ratio test (LRT). The asymptotic p-values for each voxel were computed by comparing the LRT for each voxel against the distribution and therefore signifies whether the AE model performs statistically significantly worse than the ACE model at each voxel (Supplementary Fig. S2A). For all voxels, the AE model did not perform worse than the ACE model for data representing brain activity for the fear cue greater than the safety cue. For the safety cue greater than the fear cue, we observed several voxels in which the asymptotic *p*-values were less than *p* = 0.05, and therefore we could not reject the C-component from the model (Supplementary Fig. S2B). We hence modeled data using additive heritability and unique environment and error (AE) for fear learning and with the ACE model for safety learning. Significance was calculated with 1000 permutations and cluster-based inference in the APACE software package [35] with threshold set to *p* < 0.05 based on the parametric likelihood ratio null-distribution.

#### Estimating genetic correlation between brain activation and skin conductance response

**Fig. 1:**
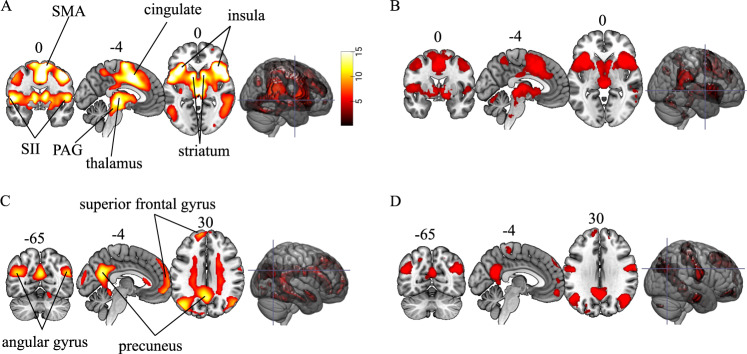
Functional activation during fear conditioning. **A** Activation map depicting brain activity during fear learning (*P* < 0.05, FWE corrected). Activated regions included bilateral insula, cingulate cortex, dorsal pons, dorsal precuneus, hypothalamus, secondary somatosensory cortex (SII), supplementary motor area (SMA), thalamus, and the ventral striatum. **B** The binary brain mask showing brain areas associated with fear learning in a meta-analysis of fear conditioning studies (Fullana et al., 2016) that was used in the present study to define a priori brain regions prior to the genetic modeling. **C** Activation map for safety learning (*P* < 0.05, FWE corrected). Activated regions included the precuneus, angular gyrus, superior frontal gyrus, middle occipital gyrus, superior medial gyrus, parahippocampal gyrus, temporal pole, lingual gyrus, and the postcentral gyrus. **D** Binary mask from Fullana et al. (2016) for the safety learning. Coordinates (*xyz*) are in Montreal Neurological Institute (MNI) space.

For each participant, mean brain activation was computed for fear and safety learning separately within distinct regions of the brain, corresponding to the masks defined in a previous meta-analysis [6] (see Fig. 1B, D). Voxel values were extracted using the python (v. 3.8.5) toolbox sklearn (v. 0.23.2) implemented in IPython (v. 7.19.0). Heritability estimates were calculated separately for mean brain activation during fear and safety learning using the mets package.

To estimate the genetic correlation between mean brain activation and SCR, we created two separate models for fear learning and safety learning. For each of the two models, mean brain activation (for fear and safety learning respectively) and SCR, representing autonomic conditioning, were used as input in OpenMx (v. 2.18.1) implemented in R (version 4.0.3). The autonomic conditioning variable was therefore the same in both models. We fitted a bivariate ACE-model and computed correlations between mean brain activation and SCR separated into A, C, and E contributions. The estimates of interest are the additive genetic influence (*h*^*2*^), the phenotypic correlation (*r*_ph_, correlation between mean brain activation and SCR), and the genetic correlations (*r*_G_) between mean brain activation and SCR. The genetic correlation describes the overlap in genetic influences on brain activity and SCR. The path diagram and bivariate model fitting can be found in Supplementary Fig. S3.

#### Estimating genetic influence on amygdala activity

The amygdala is generally considered important for fear conditioning [36–38], even though the most recent meta-analysis on human fear conditioning did not show support for increased responses to the CS+ relative the CS− [6]. Therefore, we analyzed amygdala voxels separately using AAL (automated anatomical labeling) [39] ROIs for the bilateral amygdala in WFU PickAtlas [40, 41] implemented in SPM12 (Welcome Department of Cognitive Neurology, University College, London), and estimated genetic effect using APACE [35] within the amygdala region.

## Results

We first determined the effect of fear conditioning on brain responses. We found that activated areas during fear learning (Fig. 1A) corresponded well with the brain regions described in the previous meta-analysis on fear conditioning (Fig. 1B) [6]. Safety learning also activated areas consistent with the meta-analysis [6] (Fig. 1C, D). Supplementary Tables 1 and 2 lists brain regions with peak activation during fear and safety learning. Results show that the neural circuitry engaged during fear conditioning experiments in humans is highly replicable across studies.

**Fig. 2:**
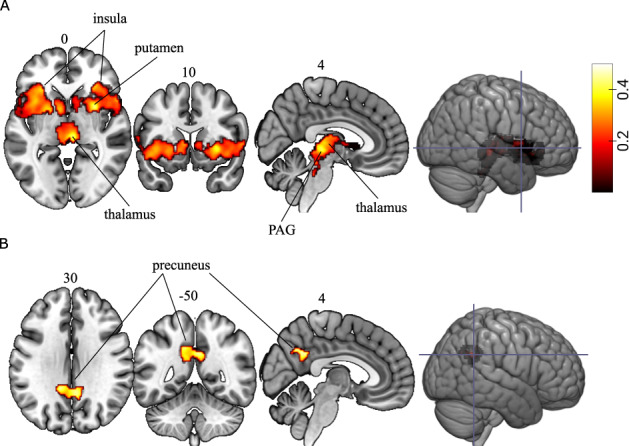
Genetic influence on brain function supporting fear conditioning. **A** The genetic influence on brain responses during fear learning and **B** safety learnine3g. Displayed voxels survived a cluster-based statistic of *P* < 0.05 family-wise error corrected. The color bar indicates genetic influence (*h*^*2*^).

We next determined genetic influences on brain activation supporting fear conditioning by decomposing the variance in brain responses into additive genetics (A) and unique environment, or error (E) [34]. We found an additive genetic influence on brain responses fear learning, in the bilateral insula, right putamen, left pallidum and right thalamus, with peak genetic effects (*h*^*2*^) ranging between 0.41 and 0.50 (Table 1 and Fig. 2A, *p* < 0.05, FWE corrected). This cluster also encompassed the periaqueductal gray of the midbrain (PAG), which is a region critical for regulating defensive responses to threat (for twin-pair correlations, see Supplementary Fig. S6). For safety learning, we could not reject the ACE model in favor for the AE model, and data was hence modeled in terms of additive genetics (A), common environment (C), and error (E). We observed genetic influences on safety learning in the range of 0.41–0.53 in a cluster encompassing the bilateral precuneus and posterior cingulate cortex (*p* = 0.05, FWE corrected, Table 1 and Fig. 2B) (see Supplementary Fig. S6 for twin-pair correlations, Supplementary Fig. S7 for the effect of common environment, *c*^*2*^, and Supplementary Fig. S8 that shows the non-overlap between the statistically significant cluster of heritability estimates and the unthresholded effect of common environment).

**Table 1:** Genetic influence, *h*^*2*^, on brain responses supporting fear conditioning.

MNI										
Contrast	Area of local maximum	MZr	DZr	*h*^*2*^	*c*^*2*^	*e*^*2*^	*X*	*Y*	*Z*	Voxels in cluster
Fear learning	R Putamen	0.56	0.12	0.50	NA	0.50	30	8	0	733
	L Insula	0.40	0.23	0.45	NA	0.55	−28	26	−4	
	L Insula	0.44	0.19	0.45	NA	0.55	−36	18	−4	
	R Inferior Frontal	0.31	0.19	0.43	NA	0.57	30	30	−10	
	R Insula	0.43	0.18	0.42	NA	0.58	42	22	−8	
	L Pallidum	0.46	0.10	0.41	NA	0.59	−20	4	−2	
	R Thalamus	0.33	0.24	0.41	NA	0.59	6	−22	6	
Safety learning	L Precuneus	0.47	0.27	0.53	0	0.47	0	-60	38	400
	Posterior cingulate cortex	0.45	0.34	0.50	0	0.50	4	−50	32	
	R Precuneus	0.38	0.27	0.49	0	0.51	6	−56	34	
	L Precuneus	0.41	0.23	0.49	0	0.51	−10	−52	38	

Unique environment and error are represented by *e*^*2*^. MZr and DZr are correlation coefficients between MZ and DZ twin pairs respectively. R = right hemisphere, L = left hemisphere.

Next, we analyzed the autonomic component of fear conditioning reflected in SCRs and computed the genetic correlation between brain activation and SCR. First, we noted that the fear cue alone elicited greater SCR’s than the safety stimuli alone, demonstrating successful fear conditioning (*t*_255_ = 23.76, *p* < 0.001). Supplementary Fig. S4 shows average SCR for the fear and safety cues. The additive genetic effect on autonomic conditioning (the difference in SCR to the fear relative to the safety cue) was *h*^*2*^ = 0.24 (CI95 = [−0.00068, 0.489], *p* = 0.002), replicating previous findings of Hettema et al. [21], of a genetic effect on fear conditioning of autonomic activity.

Next, we estimated the genetic influence on brain activity for fear and safety learning. Mean brain activation for each learning type was extracted from distinct regions of the brain corresponding to brain masks as determined in a previous meta-analysis [6] (Fig. 1B and D). The genetic influence on mean brain activation for fear learning was (*h*^*2*^ = 0.34, CI95 = [0.119, 0.562], *p* < 0.0001) and for safety learning (*h*^*2*^ = 0.54, CI95 = [0.346, 0.735], *p* < 0.0001).

For estimates of the genetic correlation between mean brain activation and autonomic conditioning, we created two separate models for fear and safety learning. The correlation (phenotypic correlation, *r*_ph_) between mean brain activation for fear learning and autonomic conditioning was *r*_ph_ = 0.19 (SE = 0.06, *z* = 2.91, *p* < 0.001) (Supplementary Fig. S5). We did not observe a statistically significant genetic correlation (*r*_*G*_ = 0.17, SE = 0.29, *z* = 0.59, *p* = 0.55). The correlation between autonomic conditioning and mean brain activation for safety learning was (*r*_ph_ = 0.14, SE = 0.07, *z* = 2.07, *p* = 0.04) (Supplementary Fig. S5). Importantly, there was a significant genetic correlation between autonomic conditioning and the mean brain activation for safety learning (*r*_G_ = 0.56, SE = 0.24, *z* = 2.34, *p* = 0.02).

Finally, because the amygdala was not part of the brain mask from the previous meta-analysis [6], yet has been indicated to have a crucial influence on fear learning in rodent studies [36–38], we computed the additive genetic effect on brain responses within the amygdala separately. We first established that the response during fear learning in the left (*t*_245_ = 8.04, *p* = 0.008, FWE corrected; MNI coordinate: *X* = −18, *Y* = 0, *Z* = −14) and right amygdala (*t*_245_ = 9.32, *p* = 0.003, FWE corrected; MNI coordinate: *X* = 20, *Y* = 2, *Z* = −14) was significant. The additive genetic effect for the same contrast within the amygdala was *h*^*2*^ = 0.32 in the left (*X* = −20, *Y* = −2, *Z* = −12), and *h*^*2*^ = 0.29 in the right (*X* = 32, *Y* = −8, *Z* = −12) amygdala, but these estimates did not survive cluster-wise inference (*p* = 0.34, FWE corrected including both hemispheres). This may reflect too low signal intensity or too high variability within the amygdalae. Thus, although we observed conditioning effects in a number of voxels in the amygdala, the genetic effect on these responses remains to be established.

## Discussion

Our results demonstrate a genetic influence on brain activity in regions supporting the acquisition of conditioned fear. The greatest additive genetic effects on fear-related activations were observed in the insula, striatum, thalamus, and PAG, while the largest effect on safety-related activation was found in an area encompassing the precuneus and posterior cingulate cortex. It is noteworthy that we found a genetic influence on mean brain activity of all voxels associated with fear conditioning recently defined in a meta-analysis [6]. This demonstrates a genetic impact across the entire circuit engaged during fear learning rather than just an influence on a activity in a few specific brain areas. We replicated the previously reported heritability for autonomic responses in fear conditioning indexed by SCR [21]. Further, we found a genetic correlation between safety-related brain activity and SCR. The general conclusion is that additive genetic influences on the neural correlates of fear conditioning are broad and affect areas responsive to the fear cue as well as areas sensitive to the safety cue.

Acquisition of conditioned fear serves as one of several models for how PTSD [42, 43] can develop. In line with the idea that fear conditioning is a model for PTSD development, experimental fear conditioning studies in patients with PTSD demonstrate stronger brain activation in several conditioning-related areas during the acquisition of conditioned fear [44]. Whether being a consequence, cause, or correlate, this indicates altered neural processing during fear conditioning in individuals that develop PTSD following trauma. However, only a fraction of all individuals experiencing trauma develop PTSD, which is consistent with the notion that genetic factors render some individuals more susceptible to remember or link traumatic experiences to environmental cues than others. Our findings are in line with this perspective, as we found genetic influences on learning strength and brain responses involved in the acquisition of conditioning. Further, the genetic influences on brain activation supporting conditioned fear were of similar magnitude as the genetic contribution to PTSD [45], as well as to the genetic influence on anxiety, as estimated in twin [21] and genome-wide association studies [46].

Acquisition of conditioned fear is a candidate mechanism for the development of certain anxiety disorders including environmentally elicited phobias [3, 4, 47, 48]. Anxiety could develop through exaggerated conditioned responses to the feared cue that has been learned to predict an aversive experience. However, the typical fear conditioning experiment (differential fear conditioning used here) also involves learning to inhibit fear to a safe control stimulus, that never predicts threat. Anxiety then could also result from impaired inhibition of fear responses to the safe cue resulting in exaggerated fear responses to environmental cues that do not signal any real danger [49]. An indication that safety learning may be an important feature for understanding anxiety comes from fear conditioning studies measuring SCR in patients with anxiety disorders. These studies show impaired safety learning across several anxiety disorders relative to controls without anxiety [50, 51]. Because these studies were performed in patients that already had developed anxiety disorders, it is not known whether reduced safety learning predisposes individuals to develop anxiety or is an effect of the disorder. The results from our study show a genetic influence on neural responses during safety learning in the precuneus and posterior cingulate cortex. Because the safety-related activation in this region was genetically influenced, it is possible that it is a predisposing factor for anxiety. We found further support for this idea, as there was a genetic correlation between mean safety-related brain activation and SCR. Because the brain controls SCR, genes that influence SCR are likely to do so by influencing brain function. An implication could be that the previously reported differences in SCR during safety learning in anxiety disorders, could be due to pre-existing differences in safety-related brain functions. As fear conditioning is a model of the acquisition of anxiety [52], the finding has implications for understanding genetic susceptibility by suggesting that genetic risk for anxiety disorders may be mediated through genetic influences on brain circuits supporting fear and safety learning.

At a more general level, fear conditioning informs on defensive responses following exposure to conditioned stressors in the environment. As such, fear conditioning can be thought of as a dimensional measure of negative valence as listed in the Research Domain Criteria of the National Institute of Health in the United States [53]. The neural signature of fear conditioning could be a phenotype of relevance for understanding brain involvement across several psychiatric disorders. It is notable that the brain regions where we observed the strongest genetic influence on fear-related activity, the insula and the striatum, corresponded to the brain regions most consistently found to show altered task-related function across anxiety disorders as well as in depression according to a large meta-analysis [54]. This suggests that fear conditioning activates a neural circuitry that is active in individuals with depression and anxiety, and future studies could evaluate whether this circuitry is associated with genetic predisposition for these disorders.

In conclusion, our findings demonstrate a genetic influence on brain responses supporting fear learning in the insula, thalamus, striatum and on brain responses supporting safety learning in precuneus. Our results highlight a neural pathway through which genes can influence aversive memory encoding and change future behavior. Findings may inform on the neural basis of genetic predisposition for stress and anxiety disorders.

## Supplementary information

Supplementary Material

## Data Availability

First-level contrast images will be made available for downloading on osf.org.

## Code availability

The code used to replicate the results can be found on the GitHub page https://github.com/granitz/twin_fear.
