# Correction: Genetic influences on central and peripheral nervous system activity during fear conditioning

**DOI:** 10.1038/s41398-022-01964-4

**Published:** 2022-05-09

**Authors:** G. Kastrati, J. Rosén, M. Fredrikson, X. Chen, R. Kuja-Halkola, H. Larsson, K. B. Jensen, F. Åhs

**Affiliations:** 1 Department of Clinical Neuroscience, Karolinska Institutet, SE-171 77 Stockholm, Sweden; 2 Department of Psychology and Social Work, Mid Sweden University, SE-831 25 Östersund, Sweden; 3 Department of Biomedical Data Sciences, Leiden University Medical Center, Leiden, the Netherlands; 4 Department of Medical Epidemiology and Biostatistics, Karolinska Institutet, SE-171 77 Stockholm, Sweden; 5 Department of Medical Sciences, Örebro University, SE-701 82 Örebro, Sweden

**Keywords:** Human behaviour, Learning and memory

Correction to: *Translational Psychiatry* 10.1038/s41398-022-01861-w, published online 08 March 2022

The original version of this article unfortunately contained a wrong label on an anatomical region. The article states that there was a statistically significant genetic influences on safety learning in midcingulate cortex in the main text and in table 1. It should be posterior cingulate cortex and not midcingulate cortex. The authors apologize for the error. The original article has been corrected.

