# Supplementary material for: Genetic influences on central and peripheral nervous system activity during fear conditioning
Source: Transl Psychiatry. 2022 Mar 8;12:95. doi: 10.1038/s41398-022-01861-w (PMC8904491; doi:10.1038/s41398-022-01861-w)
Supplement: Supplementary file 1 — Supplementary Material [file 41398_2022_1861_MOESM1_ESM.docx]

Supplementary Materials for

**Genetic influences on central and peripheral nervous system activity during fear**G. Kastrati^1,6^*, J. Rosén ^2^, M. Fredrikson^1^, X. Chen^3^, R. Kuja-Halkola^4^, H. Larsson^5^., K.B. Jensen ^1^, F. Åhs^6^.

*Correspondence to: granit.kastrati@ki.se

**List of Supplementary Materials:**

Supplementary Table S1.

Supplementary Table S2.

Fig. S1.

Fig. S2.

Fig. S3.

Fig. S4.

Fig. S5.

Fig. S6.

Fig. S7.

Fig. S8.

*Functional MRI Data and SCR Outlier Identification*

We identified univariate outliers in our data sample using the median absolute deviation (MAD) method (*1*). For everyone, we computed the mean contrast value of all brain voxels for each first-level contrast. Participants with a mean contrast value deviating more than 3 times the median standard deviation were removed as well as their co-twins. Included in the final twin-analysis of the contrast images for fear and safety learning was a sample of 56 MZ (35 female, 21 male) and 67 DZ (39 female, 28 male) twin pairs. For the genetic modeling of SCR, we identified outliers in our data the same way as above. Removal of outliers was performed to increase the robustness of the correlation between MZ and DZ twins, and hence *h^2^* estimates. Participants with a SCR to the fear cue greater than the safety cue deviating more than 3 median absolute deviations from the median were excluded. Using this method, three participants were excluded as well as their co-twin. Additionally, SCR data was missing from one twin which was excluded from analysis together with the co-twin. Included in the final statistical analysis of SCR data was a sample of 62 MZ (35 female, 27 male) and 66 DZ twin pairs (38 female, 28 male).

**Supplementary Table 1** Brain areas activated during fear-learning (*P <0.05, family-wise error corrected*). R = right hemisphere, L = left hemisphere.

**Supplementary Table 2**. Brain areas activated during safety-learning (*P <0.05, FWE*). R = right hemisphere, L = left hemisphere.

**Fig. S1**. Experimental design. Two virtual characters served as conditioned stimuli (CS). One of the virtual characters served as fear cue (CS+) and predicted the unconditioned stimulus (US) whereas the other character served as safety cue (CS-) and was never associated with the US. Each CS-type appeared 16 times each for 6s with an inter-stimulus interval of 8-12s. Eight of the CS+ presentations co-terminated with presentation of the US (50% reinforcement schedule). Four stimulus presentation orders were used to counterbalance CS across subjects. Prior to the experiment, participants were told that they could learn to predict the US but were not told which character served as CS+.

**Fig. S2**. Distribution of asymptotic p-values. A p-value for a voxel signifies whether the AE model performs statistically significantly worse than the ACE model. (A) The asymptotic p-values for brain responses to fear learning and for (B) the safety learning. The vertical lines denote the point where p = 0.05.

**** **Fig. S3**. Standardized path diagram showing the estimated additive genetic influence on autonomic conditioning (A1) and mean neural response (A2). The bivariate correlation between mean neural response and autonomic conditioning is also described. The models were fitted to mean contrast values within the a priori brain masks related to (A) fear learning and (B) safety learning. The bivariate model fit for fear learning using the Akaike Information Criterion was AIC = -207.4, for the AE model, and -201.4 for the ACE model. The corresponding fit index for safety learning was AIC = -206.0 for AE model, and AIC = -200.0 ACE model.


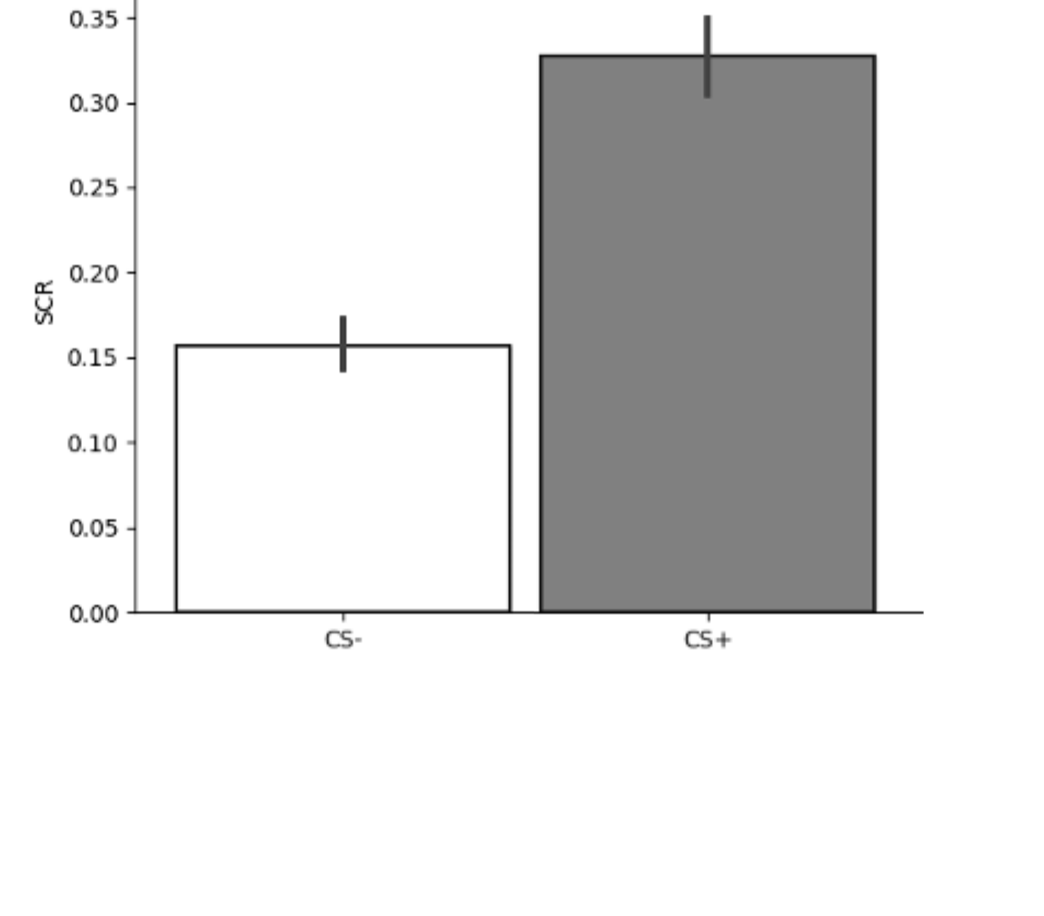


**Fig. S4.** Skin conductance responses for the fear (CS+) and safety cue (CS-).

**
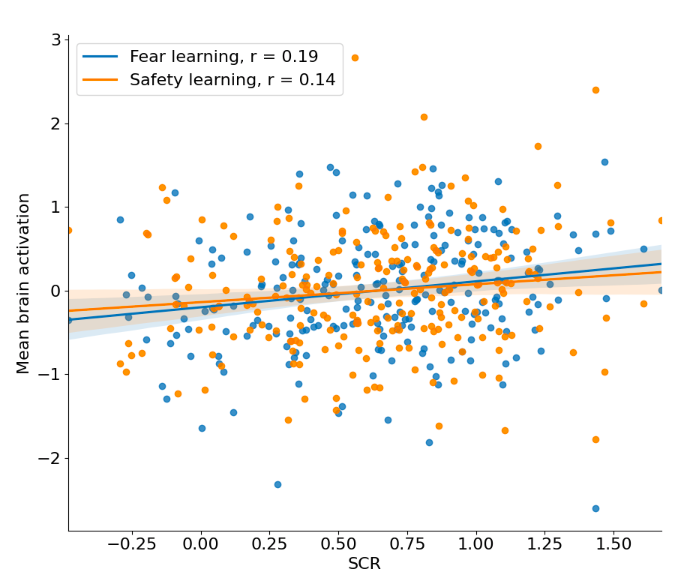
**

**Fig. S5**. Scatter plot of mean brain activation and skin conductance response (SCR). Mean brain activation was computed separately from voxels within regions predetermined by the brain mask depicted in Figure 3B (blue color) and Figure 3D (orange color) based on the meta-analysis by Fullana et al. (2016).

**Fig. S6**.  Between twins correlations. Correlation for fear learning for (**A**) MZ and (**B**) DZ twins. Correlations for safety learning for (**C**) MZ and (**D**) DZ twins. Correlations are displayed within the same regions as in Fig. 1B and D. The color bar indicates correlation (*r*). MZ = monozygotic, DZ = dizygotic.

**Fig. S7**. The effect of common environment *c^2^* on brain responses to safety learning.


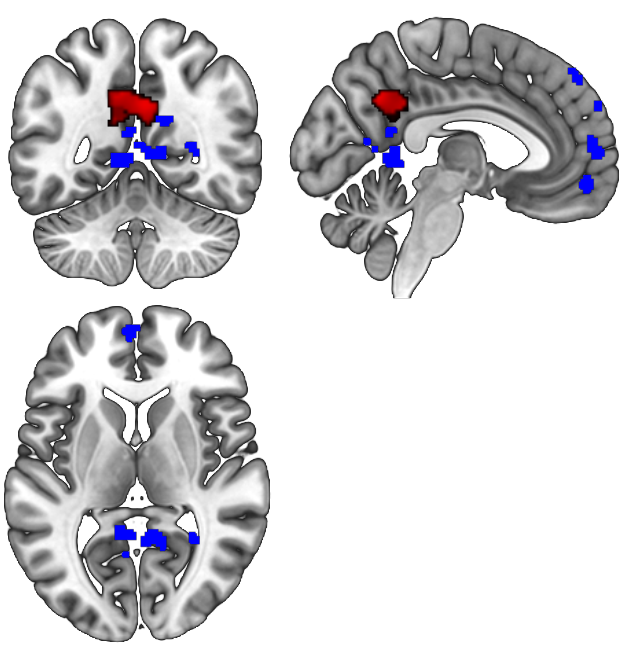
 **Fig. S8**. Effects of shared environment on brain responses during safety learning. Unthresholded estimates of shared environment (*c^2^*) displayed in blue on an anatomical template brain (same estimates as in Supplementary Fig. S7). The significant cluster of voxels showing an additive genetic influence (*a^2^*) on brain responses during safety learning is displayed in red. There was no overlap between clusters showing a genetic (red) and shared environmental (blue) influence on brain responses during safety learning.

References and Notes:

1. C. Leys, C. Ley, O. Klein, P. Bernard, L. Licata, Detecting outliers: Do not use standard deviation around the mean, use absolute deviation around the median. *J Exp Soc Psychol* **49**, 764-766 (2013).
